# Effects of Early Postoperative Whey Protein Isolate-Based Oral Supplementation on Nutritional Outcomes in Patients Undergoing Coronary Artery Bypass Grafting: A Prospective Multicentre Real-World Study

**DOI:** 10.7759/cureus.115936

**Published:** 2026-09-08

**Authors:** Haritha Shyam, Ritika Samaddar, Rajesh K Chand, Jana D Gandivalasa, Somya Shrivastava, Sejal Patel, Chetna Shah

**Affiliations:** 1 Dietetics, Apollo Hospitals, Hyderabad, IND; 2 Dietetics, Max Healthcare, New Delhi, IND; 3 Cardiac Anesthesia, Max Healthcare, New Delhi, IND; 4 Medical Affairs, Otsuka Pharmaceutical India Pvt. Ltd., Ahmedabad, IND

**Keywords:** early postoperative nutrition, lean body mass, muscle strength, oral nutritional supplement, protein, undernutrition, whey protein isolate

## Abstract

Background

Coronary artery bypass grafting (CABG) triggers inflammatory and catabolic responses, increasing the risk of postoperative nutritional deficits. Whey protein isolate (WPI) may improve recovery and muscle strength. This study evaluated the impact of early postoperative WPI-based supplementation on nutritional outcomes among CABG patients.

Methodology

This open-label, single-arm, prospective real-world study enrolled 200 adult patients undergoing CABG at two tertiary care centres in India. Patients received a WPI-based oral nutritional supplement starting 24-48 hours post-surgery and continued for 30 days post-discharge. Outcomes included protein and calorie adequacy, nutritional status, change in weight, hand-grip strength, safety, and patient and physician experiences with the WPI-based nutritional supplement. Data were analysed using appropriate parametric and nonparametric tests.

Results

Of the 200 patients enrolled, 182 (91.0%) completed the study. They were included in the intent-to-treat (ITT) group, while 119 (59.5%) adhered to the protocol, completed the 30-day follow-up visit, and were included in the per-protocol (PP) group. Compared with the day of supplement initiation (Visit 1), the proportion of patients achieving calorie and protein adequacy at the end of the study (EOS) increased significantly (calorie adequacy: ITT (*n* = 34, 18.7%) vs. (*n* = 143, 81.3%); PP (*n* = 3, 2.5%) vs. (*n* = 105, 88.2%); protein adequacy: ITT (*n* = 41, 22.5%) vs. (*n* = 140, 76.9%); PP (*n* = 5, 5.0%) vs. (*n* = 99, 83.2%); all *P* < 0.001). An increase in weight was observed in both groups (*P* < 0.05), with a greater proportion of patients experiencing weight gain in the PP group (*n* = 60, 50.0%) than in the ITT group (*n* = 55, 30.0%) at EOS. Hand-grip strength (HGS) also improved from the day of discharge (Visit 3) to EOS (HGS weak: ITT (*n* = 130, 71.4%) vs. (*n* = 71, 39.0%) (*P* < 0.001); PP (*n* = 85, 71.4%) vs. (*n* = 38, 31.9%) (*P* < 0.001); HGS normal: ITT (*n* = 43, 23.6%) vs. (*n* = 85, 46.7%) (*P* < 0.001); PP (*n* = 31, 26.1%) vs. (*n* = 65, 54.6%) (*P* < 0.001); HGS strong: ITT (*n* = 9, 5.0%) vs. (*n* = 26, 14.3%) (*P* < 0.05); PP (*n* = 3, 2.5%) vs. (*n* = 16, 13.5%) (*P* < 0.05)). No related safety events were recorded.

Conclusions

Early WPI-based oral supplementation initiated within 48 hours post-CABG and continued for 30 days increases protein and calorie adequacy, nutritional outcomes, supported weight gain, and supported muscle strength recovery without safety concerns. These findings support incorporating early protein supplementation into postoperative care protocols.

## Introduction

Undernutrition is a prevalent yet under-recognised concern among cardiac surgery patients. It is often exacerbated postoperatively by surgical stress, systemic inflammation, and delayed nutritional intervention [1,2]. Approximately 1.2%-46.4% of patients subjected to cardiac surgery are undernourished [1]. Coronary artery bypass grafting (CABG), a major surgical procedure, induces inflammatory and catabolic stress responses, increasing susceptibility to perioperative nutritional deficiencies [3]. Postoperatively, body composition changes markedly, with an average 5% reduction in body weight, along with reductions in lean body mass (LBM) and skeletal muscle mass. These changes are associated with impaired functional recovery and an elevated risk of morbidity, mortality, and prolonged hospitalisation [4]. Patients with low LBM and fat-free mass, particularly those undergoing cardiac surgery, exhibit diminished physiological reserves, rendering them less able to withstand the metabolic stress of recovery and thereby increasing their susceptibility to postoperative complications such as infection, delayed wound healing, and impaired rehabilitation [5].

Nutritional support is an important component of perioperative care and may contribute to improved postoperative recovery among cardiac surgery patients [1]. Well-nourished patients consistently demonstrate improved recovery and reduced mortality following cardiac surgery [6]. Evidence from global cardiac surgery literature suggests that nutritional support may be suboptimal in some postoperative cardiac surgery settings, highlighting an important gap in perioperative nutrition care [6, 7]. In a study by Rahman et al. involving patients undergoing CABG surgery from ICU admission to ICU discharge, 40% of post-CABG ICU patients received no nutritional support, and enteral nutrition was initiated after a mean of 2.3 ± 1.8 days [8].

Clinical guidelines from the European Society for Clinical Nutrition and Metabolism (ESPEN) and the Enhanced Recovery After Surgery (ERAS) protocols provide structured, evidence-based recommendations to optimise perioperative nutritional care and support recovery after surgery, including reduction of postoperative complications, hospital length of stay, healthcare costs, and mortality [2,9-11]. ESPEN guidelines recommend initiating early enteral nutrition within 24 hours postoperatively, with energy and protein targets of 25-30 kcal/kg/day and up to 1.5 g/kg/day of ideal body weight, respectively, for surgical patients [7,9,12]. Additionally, in line with the ESPEN guidelines, the Scientific Statement by the American Heart Association recommends enteral nutrition as the preferred mode of postoperative nutritional support, with initiation within 48 hours of surgery or within 24 hours in malnourished patients. Initial feeding should be hypocaloric, providing approximately 80% of targeted calorie and protein requirements. Protein targets of 1.0 to 1.2 g/kg/day may be suitable for patients with cardiac cachexia, while current guidelines for critically ill adults recommend 1.2 to 2.0 g/kg/day [7,8,13].

Protein constitutes a critical component of nutritional support, playing a pivotal role in optimising clinical outcomes [14,15]. Adequate protein intake is vital for surgical recovery, as it supports modulation of the stress response, preserving muscle function, wound healing, and functional recovery. However, studies show that postoperative patients often consume only 22%-36% of their recommended protein intake. Suboptimal protein intake during this period may increase the risk of muscle atrophy, impaired wound healing, and delayed functional recovery [16]. Leucine, an essential branched-chain amino acid (BCAA), activates the mechanistic target of rapamycin (mTOR) pathway, thereby facilitating muscle protein synthesis and supporting the maintenance of LBM [17,18]. Evidence from cardiac rehabilitation settings suggests that leucine supplementation may significantly improve protein intake and LBM after six months [19]. Additionally, leucine-enriched protein supplementation has been associated with improvement in muscle and grip strength and functional recovery [19].

Given the high metabolic demands and risk of suboptimal protein intake during surgical recovery, use of a high-quality protein supplement such as whey protein isolate (WPI) may help support postoperative nutritional requirements [16,17,20]. WPI is rapidly absorbed, contains high leucine content (8.6 g/100 g), and has minimal lactose and fat. WPI has better bioavailability and anabolic effectiveness than casein [21,22]. Its refined composition (>90% protein, minimal lactose and fat content) makes it suitable for surgical patients with compromised digestion or inflammatory states. While whey protein concentrates also contain essential amino acids, their comparatively lower protein content (35%-80%) and higher lactose content may limit their use in sensitive populations [17,18]. Beyond its amino acid profile, WPI also contains bioactive compounds - viz., immunoglobulins and glycomacropeptides - that support immune function, antioxidant defence and exhibit antimicrobial activity [17].

Emerging evidence suggests WPI may accelerate recovery, shorten hospital stay, and maintain nutritional status in perioperative patients. Studies in patients with chronic heart failure have shown that WPI supplementation may improve body composition by increasing skeletal muscle mass and reducing fat mass [23,24]. However, evidence demonstrating similar effects specifically in patients undergoing CABG remains limited.

Despite growing awareness of the role of protein and oral nutritional supplements in postoperative recovery, data specifically evaluating early postoperative WPI-based nutritional supplementation in patients undergoing CABG remain limited. This prospective, multicentre, real-world study evaluates the impact of early WPI-based nutrition supplements on calorie and protein adequacy, hand-grip strength, and changes in body weight among post-CABG patients. The study also assesses the safety and tolerability of WPI supplementation in a real-world setting. The article was previously presented as a conference abstract at the 2026 International Conference on Clinical Nutrition (ICNC) on February 8, 2026.

## Materials and methods

Study design

This was a prospective, open-label, single-arm observational study conducted in a real-world clinical practice setting to evaluate the effectiveness and safety of a WPI-based oral nutritional supplement in patients undergoing CABG. The study was conducted in a real-world setting, and blinding was not used. Patients were screened according to predefined inclusion and exclusion criteria and enrolled if they were deemed suitable for postoperative supplementation based on the investigator's assessment and the patient's nutritional requirements. WPI-based nutritional supplementation was prescribed at the investigator’s discretion based on each patient’s postoperative nutritional requirements and oral dietary intake, consistent with routine clinical care.

A total of 200 patients were enrolled at two study sites in India, viz., Apollo Hospital in Hyderabad (*n* = 125) and Max Hospital in New Delhi (*n* = 75). Of these, 182 patients (91.0%) were included in the intent-to-treat (ITT) group, 119 (59.5%) were included in the per-protocol (PP) group, and 18 (9.0%) did not complete the study. Patients were followed up by designated dietitians at the predetermined time points, and the corresponding data were collected at these visits. The dietitians also ensured that all relevant study documents were maintained.

Eligible patients received the WPI-based nutritional supplement (2-6 scoops) for 30 consecutive days to meet their daily protein requirements. The product contains WPI as the protein source, with each serving (2 scoops) providing 11.3 g of protein and 89 kcal. The recommended supplement intake was individualised by the investigators based on each patient’s nutritional requirements and oral dietary intake. The detailed composition of the WPI-based nutritional supplement is provided in Table 1. The study sponsor is the manufacturer of the nutritional supplement under investigation. However, the sponsor had no role in patient selection, data collection, or interpretation of the results.

**Table 1:** Detailed composition of the whey protein isolate nutritional supplement used in the study. Note: %RDA: Value established according to ICMR 2020 guidelines (men, sedentary work); (-): RDA value not established. Ingredients: Whey protein isolate, maltodextrin, inulin, minerals, vanilla flavour, stabiliser: xanthan gum (INS 415), vitamins, choline bitartrate. The product is commercially available as HINEX HP and was manufactured by Otsuka Pharmaceutical India Pharma Limited, headquartered in Ahmedabad, India. ICMR, Indian Council of Medical Research; NMT, not more than; RDA, recommended dietary allowance; RE, retinol equivalents; TE, tocopherol equivalents

Product composition	Units	Per 100 g powder	Per 25 g serving	% RDA per serving
Energy	kcal	356	89	4
Protein	g	45.0	11.3	21
Carbohydrate	g	45.4	11.4	9
Total sugars	g	1.7	0.43	-
Added sugars	g	0.00	0.00	-
Dietary fibre (Prebiotic)	g	5	1.25	4
Fat, NMT	g	1.5	0.38	1
Saturated fat, NMT	g	0.8	0.20	1
Trans-fatty acids, NMT	g	0.02	0.005	0.3
Cholesterol	mg	6	1.5	-
Choline	mg	100	25	-
Vitamins				
Vitamin A (RE)	mcg	500	125	13
Vitamin D2	mcg	8.0	2.0	13
Vitamin E (Alfa TE)	mg	7.5	1.9	19
Vitamin K1	mcg	45	11	20
Vitamin C	mg	75	19	23
Vitamin B1	mg	1.6	0.40	29
Vitamin B2	mg	1.6	0.40	20
Vitamin B6	mg	1.6	0.40	21
Vitamin B12	mcg	2.3	0.58	26
Niacin	mg	9.0	2.3	16
Folic acid	mcg	150	38	21
Pantothenic acid	mg	3.0	0.75	15
Biotin	mcg	25	6.3	16
Minerals				
Sodium	mg	80	20	1
Potassium	mg	490	123	4
Chloride	mg	280	70	3
Calcium	mg	720	180	18
Phosphorus	mg	275	69	7
Magnesium	mg	125	31	7
Iron	mg	4.0	1.0	5
Zinc	mg	5.0	1.3	7
Manganese	mg	1.5	0.38	9
Copper	mcg	500	125	7
Iodine	mcg	100	25	18
Selenium	mcg	30	7.5	19
Chromium	mcg	35	8.8	18
Molybdenum	mcg	40	10.0	22

Study population

The inclusion criteria comprised male and female patients aged 18-70 years with a confirmed diagnosis of coronary artery disease who were scheduled to undergo CABG and were suitable to receive WPI supplementation within 24-48 hours after surgery. Patients were required to have a minimum hospital stay of five days, including ICU admission, and to receive standard nutritional care. Additionally, patients were required to adhere to the study visit schedule and other protocol requirements. Each patient was assigned a unique patient number for identification throughout the study. The study was conducted in accordance with the ethical principles of the Declaration of Helsinki, and written informed consent was obtained from all participants before enrolment.

The major exclusion criteria included the use of enteral or parenteral nutritional supplements or any other form of protein supplement; known allergies or contraindications to WPI-based nutritional supplements; pregnancy or breastfeeding; body mass index (BMI) >30 kg/m²; severe or life-threatening complications or a likelihood of not surviving the next 48-72 hours at the time of enrolment; use of other experimental drugs or participation in another clinical trial within the preceding month; and any clinical condition that, in the investigator's opinion, precluded participation in the study.

For statistical analysis, patients were categorised into analysis populations. The ITT population included all enrolled patients, regardless of protocol adherence, to account for variability in visit timing and potential protocol deviations. Patients who were enrolled but did not complete the study had insufficient outcome data for evaluation of the primary and secondary endpoints. Therefore, analyses were conducted in the evaluable population, comprising all enrolled patients with adequate post-baseline data for endpoint assessment. The PP population comprised patients who adhered to the study protocol, including compliance with the specified study timelines and procedures.

Ethics approval and consent to participate

This study was approved by the Institutional Ethics Committee (IEC) at both sites: Apollo Hospitals, Hyderabad (EC Reg. No: ECR/38/Inst/AP/2013/RR-19; NABH Certificate No.: EC-CT-2018-0037; IEC Application No: AHJ-CS-018/11-22) and Devki Devi Foundation, New Delhi (Registration No.: EC/NEW/INST/2023/DL/0220; Study IEC Ref. No.: BHR/RS/MSSH/DDF/SKT2/IEC/CND/23-06). The ethics review board members were independent of the investigators and sponsor. The real-world study adhered to national guidelines and the Declaration of Helsinki. The informed consent form (ICF) was obtained from all participants before enrolment. The study was registered prospectively on 22 August 2023 with the Clinical Trials Registry-India (CTRI/2023/08/056773) (www.ctri.nic.in).

Stages of study

The study included a screening period (up to three days) from the day of CABG surgery and 30 days from the start of the WPI-based nutritional supplement.

Screening Period

After consent, patients were screened for eligibility and underwent baseline assessments. WPI supplementation commenced 24-48 hours post-surgery, as determined by the investigator.

Treatment Period

Patients received 2-6 scoops of WPI-based oral supplement daily for 30 days, based on daily caloric and protein requirements determined by the investigators, in addition to standard nutritional care. Nutritional intake was individualised based on the investigator's assessment of each patient's nutritional status and requirements using actual body weight, targeting 25-30 kcal/kg/day of energy and 1.1-1.5 g/kg/day of protein from the combined oral diet and WPI-based nutritional supplement, in accordance with ESPEN guideline recommendations. Two scoops of the nutritional supplement provided 11.3 g of protein and 89 kcal of energy. Accordingly, patients were prescribed 2-6 scoops based on their requirements and ability to take an oral diet. Patients with adequate oral intake generally received 2-4 scoops, whereas patients with insufficient oral intake were prescribed 5-6 scoops to help maintain nutritional status during the postoperative recovery period. The study also complied with ERAS principles, and supplementation was initiated within 24-48 hours post-surgery. Oral intake was assessed using patient/caretaker-maintained dietary intake records documented in the patient diary and dietary card. Daily oral food intake and WPI-based supplement consumption were recorded throughout the study period and reviewed by the hospital study staff/investigator during follow-up visits to estimate oral intake and assess nutritional adequacy.

Follow-up Assessment

Effectiveness and safety were evaluated during hospitalisation and at follow-up visits. Data were collected at baseline (screening visit), Visit 1 (24-48 hours post-surgery and initiation of the WPI-based oral nutritional supplement), Visit 2 (transfer from the ICU to the ward), Visit 3 (hospital discharge), and Visit 4 (end of study [EOS]: 30 days of supplementation post-discharge).

Study assessments

Given the real-world observational design, no formal hypothesis-driven sample size calculation was performed. The planned enrolment of 200 patients was based on feasibility, anticipated attrition, and the need for an evaluable cohort large enough to describe nutritional outcomes in routine clinical practice. Assuming approximately 10%-15% attrition, 200 enrolled patients were expected to yield approximately 170-180 evaluable patients. A sample size of 200 also provides acceptable precision for estimating key binary outcomes, with an approximate two-sided 95% confidence interval margin of error of ±7% under the conservative assumption of a 50% event proportion.

Baseline data included patient demographics, medical and surgical history, and prior treatment history. The primary endpoints of the study included adequacy of total calorie and protein intake, anthropometric measurements (body weight), and hand-grip strength (average of three trials using a hand dynamometer). Calorie and protein adequacy were defined as achieving at least 80% of the prescribed daily calorie and protein targets, respectively. Evidence suggests that achieving 80% of the prescribed calorie or energy intake may be optimal during the acute phase of hospitalisation [25,26]. Secondary endpoints included nutritional status using the Subjective Global Assessment [27], safety assessment, and patient and physician experience with the WPI-based nutritional supplement. Patient compliance and feedback regarding the WPI-based nutritional supplement were recorded, along with the physician's observations and experience.

Baseline nutritional intake (at the time of initiation of the nutritional supplement) was recorded and reported. The study sites followed the nutritional target as defined; hence, no major differences in the conduct of the study were observed. Achievement of calorie and protein requirements was categorised as adequate or inadequate according to prespecified nutritional targets and analysed as binary outcomes. Hand-grip strength was measured using a hand dynamometer. For categorical analyses, participants were classified into weak, normal, or strong hand-grip strength categories according to predefined reference thresholds. Changes in the distribution of these categories over time were assessed using non-parametric methods. Supplement adherence and compliance were assessed using patient/caretaker-maintained diaries and dietary cards issued at discharge. Patients or caretakers recorded daily food intake and WPI-based supplement consumption until the end of the study. These records were reviewed by the hospital study staff/investigator during follow-up visits. Physician experience with WPI-based supplementation was assessed at the end of the study using a structured questionnaire, which captured the physician's overall experience, including ease of use, tolerability, and suitability as part of postoperative nutritional care. Adverse events were monitored and documented from the day the ICF was signed through EOS.

Routine clinical practice was preserved with respect to patient selection, nutritional assessment, dietary counselling, supplement dose recommendation, and postoperative management. The supplement was already used as part of routine care at both sites and was not specifically introduced for the study. WPI-based supplement intake was individualised by the investigators based on each patient's nutritional requirements and oral dietary intake, while dietary counselling and postoperative care followed standard hospital protocols. The study protocol specified follow-up visits and outcome assessments for systematic data collection only; no additional protocol-mandated interventions were imposed beyond routine clinical care.

Statistical analysis

The patients were segregated into ITT and PP groups, and the endpoints were assessed for both groups. The ITT analysis reflected the real-world clinical setting, where variability in patient behaviour, protocol adherence, and incomplete data is common. It provided a realistic and comprehensive estimate of the treatment effect, enhancing the applicability and external validity of the study results. However, the findings should be interpreted with caution since the ITT group only included patients who completed the study. The PP analysis focused on complete cases, excluding those with significant protocol deviations or missing data.

Demographic and baseline characteristics were summarised using standard descriptive statistics. Continuous variables were described using the number of non-missing observations (*n*), means, standard deviations (SDs), medians, ranges, and the corresponding 95% confidence intervals (CIs). Categorical variables were presented as frequencies and proportions. For longitudinal assessments involving multiple study visits, an overall repeated-measures analysis was performed before pairwise comparisons. Binary outcomes were evaluated using Cochran's Q test, continuous outcomes using repeated-measures ANOVA (or non-parametric alternatives when distributional assumptions were not met), and ordinal outcomes using the Friedman test. When a significant overall difference was identified, pairwise comparisons between visits were conducted using the appropriate post hoc tests. Bonferroni correction was applied to control the family-wise error rate resulting from multiple comparisons.

Calorie and protein adequacy were classified as binary variables (adequate/inadequate) at each study visit based on predefined nutritional targets. Changes in the proportion of patients achieving adequacy across visits (Visit 1, Visit 3, and EOS) were evaluated using Cochran's Q test, followed by pairwise McNemar tests where appropriate. The Shapiro-Wilk test was used to assess the normality of body weight measurements. As the data were found to be normally distributed, changes in body weight across study visits were evaluated using repeated-measures analysis of variance (ANOVA), with paired t-tests performed for pairwise comparisons between visits where appropriate (Baseline, Visit 1, Visit 3, and EOS). Hand-grip strength categories (weak, normal, strong) were compared across visits using the Friedman test. Pairwise comparisons were performed using Wilcoxon signed-rank tests with adjustment for multiple comparisons. McNemar's test was used only for paired analyses of categorical transitions between hand-grip strength categories across visits. All statistical tests were performed at the 5% level of significance, defined as P < 0.05. All statistical analyses were conducted using R statistical software (version 4.5.1; R Foundation for Statistical Computing, Vienna, Austria, 2025).

As this was a single-arm observational study, primary analyses were based on within-patient changes over time, and no multivariable adjustment was performed for baseline covariates such as age, sex, diabetes status, baseline BMI, baseline nutritional status, or study site.

Missing data were handled according to the prespecified Statistical Analysis Plan. For the ITT population, missing post-baseline observations were imputed using the Last Observation Carried Forward (LOCF) method. Under this approach, the last available post-baseline assessment for a participant was carried forward to replace subsequent missing values. The PP analysis included patients with evaluable outcome data and no major protocol deviations.

## Results

Patient characteristics

Of the 200 patients enrolled, 182 patients completed the study and were included in the ITT group (*n* = 182, 91.0%) (Figure 1). Among these, 119 patients adhered to the study protocol and completed the required follow-up assessments, forming the per-protocol (PP) group (*n* = 119, 59.5%). The remaining patients (*n* = 63, 40.5%) had an incomplete follow-up duration (either early, *n* = 40, 20.0%, or post, *n* = 23, 11.5%, predefined time point) and were included in the ITT group. A total of 18 patients did not complete the study (*n* = 18, 9.0%).

**Figure 1:**
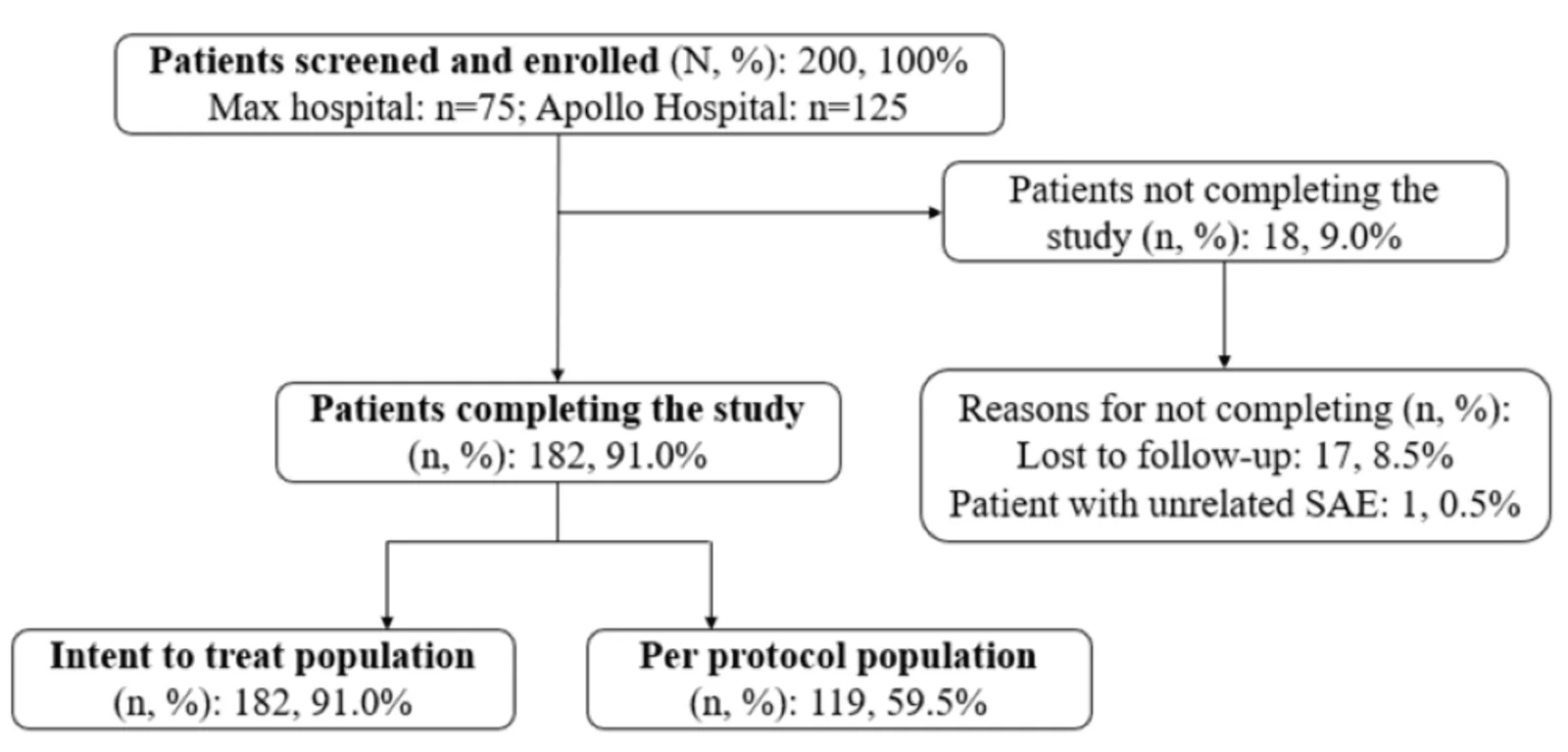
Overall patient disposition for the study. The figure was created in Microsoft Word, and pixels were adjusted using Microsoft Paint (Microsoft Corporation, Redmond, WA). SAE, serious adverse event; *N*, total number of patients; *n*, number of patients

The mean age of patients in the ITT and PP groups was 57.9 ± 8.3 and 56.9 ± 7.5 years, respectively. A male preponderance was observed (ITT: *n* = 174, 95.6%; PP: *n* = 113, 95.0%). The mean height was 164.1 ± 8.4 cm (ITT group) and 162.4 ± 8.5 cm (PP group), while the mean weight was 65.5 ± 9.9 kg (ITT group) and 64.9 ± 8.9 kg (PP group) (Table 2). The corresponding BMIs for the ITT and PP groups were 24.7 ± 2.7 and 24.6 ± 8.9 kg/m², respectively.

**Table 2:** Baseline characteristics of the patients included in the study. Demographic and baseline characteristics were summarised using standard descriptive statistics. Continuous variables were described using the number of non-missing observations (n), means, and SDs. Categorical variables were presented as frequencies and proportions. BMI, body mass index; ITT, intent-to-treat; N, total number of patients; PP, per-protocol; SD, standard deviation

Characteristics	Population	
	ITT (*N* = 182)	PP (*N* = 119)
Percentage of patients	100.0%	100.0%
Age (years)		
Mean age	57.9	56.9
SD	8.3	7.5
Gender		
Male, *n* (%)	174 (95.6)	113 (95)
Female, *n* (%)	8 (4.4)	6 (5)
Height (cm)		
Mean	164.1	162.4
SD	8.4	8.5
Weight (kg)		
Mean	66.5	64.9
SD	9.9	8.9
BMI (kg/m^2^)		
Mean	24.7	24.6
SD	2.7	2.2

Calorie and protein adequacy

The nutritional target was 25-30 kcal/kg/day of calories and 1.1-1.5 g/kg/day of protein from the oral diet and a WPI-based nutritional supplement, in line with the ESPEN guideline recommendation. Both the ITT and PP groups showed an increase in calorie and protein adequacy from Visit 1 (day of initiation of the supplement) to EOS. In the ITT group, the proportion of patients with calorie adequacy increased from 34 (18.7%) at Visit 1 to 148 (81.3%) at EOS (*P* < 0.001), while in the PP group, it increased from 3 (2.5%) at Visit 1 to 105 (88.2%) at EOS (*P* < 0.001) (Figure 2). Protein adequacy increased from 41 (22.5%) at Visit 1 to 140 (76.9%) at EOS in the ITT group (*P* < 0.001), and from 6 (5.0%) at Visit 1 to 99 (83.2%) at EOS in the PP group (*P* < 0.001) (Figure 2). On comparing the PP and ITT groups, the PP group demonstrated an increase in both protein (*n* = 99, 83.2%, vs. n = 140, 76.9%) and calorie intake (*n* = 105, 88.2%, vs. *n* = 148, 81.3%) at EOS. The pairwise statistical comparison is provided in Table 3.

**Figure 2:**
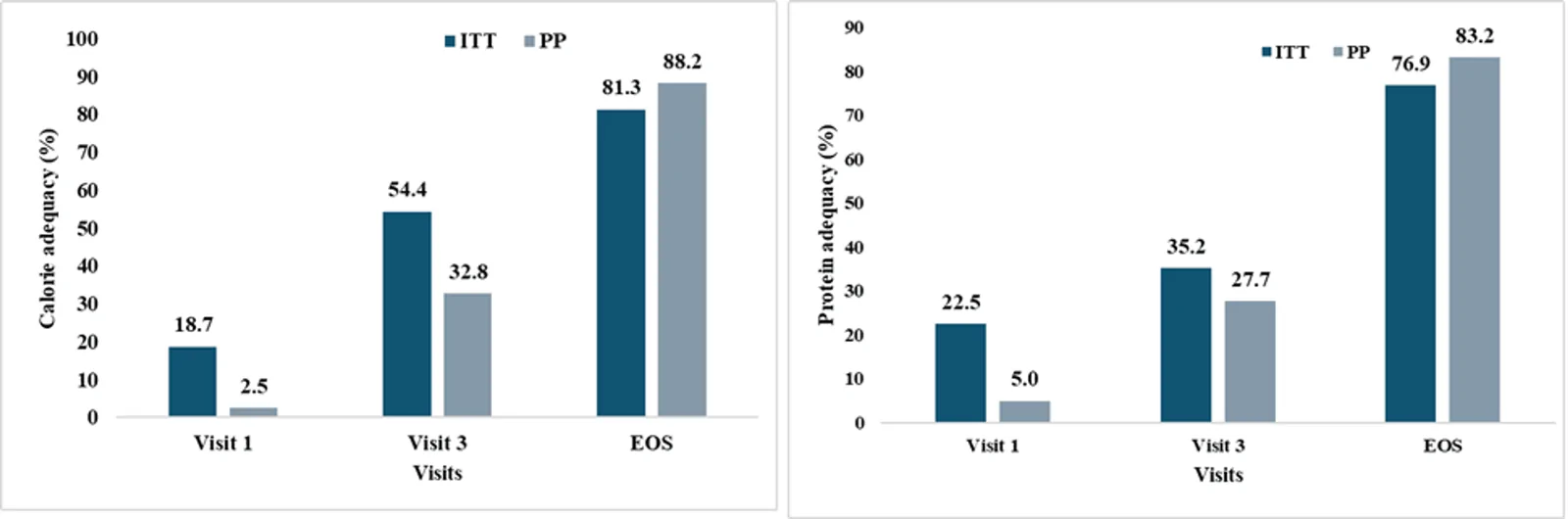
Summary of calorie and protein adequacy achieved. Cochran's Q test was employed to assess the differences in the proportion of patients achieving adequate calorie and protein intake across all study visits. For both groups, the *P*-value was less than 0.05, indicating that the observed differences in calorie and protein intake adequacy across visits are unlikely to be due to random chance and are more likely the result of the intervention or treatment. Number of patients in the ITT group: *N* = 182 (100.0%); number of patients in the PP group: *N* = 119 (100.0%). The figure was prepared in Microsoft Excel, and pixels were adjusted using Microsoft Paint (Microsoft Corporation, Redmond, WA). Visit 1 = 24-48 hours post-surgery and initiation of WPI-based oral nutritional supplement; Visit 3 = hospital discharge; and EOS = 30 days of supplementation post-discharge. ITT, intent-to-treat; PP, per-protocol; EOS, end of study

**Table 3:** Comparative assessment of calorie and protein adequacy between visits in the ITT (N = 182) and PP (N = 119) groups. Note: McNemar's test was used to evaluate changes in calorie and protein adequacy through pairwise comparisons across study visits. Statistically significant differences were observed for all visit comparisons (Visit 1 vs. Visit 3, Visit 1 vs. EOS, and Visit 3 vs. EOS) in both the ITT and PP populations. Statistical significance was assessed using a predefined alpha level of 0.05, with *P*-values < 0.05 considered significant and *P*-values ≥ 0.05 considered non-significant. Similarly, adjusted *P*-values were interpreted using the same threshold, with adjusted significance (p.adj.signif) classified as *Yes* for *P*-values < 0.05 and *No *for *P*-values ≥ 0.05. Number of patients in the ITT group: *N* = 182, 100.0%; number of patients in the PP group: *N* = 119, 100.0%. Visit 1 = 24-48 hours post-surgery and initiation of WPI-based oral nutritional supplement; Visit 3 = hospital discharge; and EOS = 30 days of supplementation post-discharge. EOS, end of study; ITT, intent-to-treat; PP, per-protocol; df, degrees of freedom; p.adj, adjusted *P*-value

Pairwise comparison		Test statistic (χ²)		*P*-value		p.adj		df		p.adj.signif (Yes/No)	
Schedule 1	Schedule 2	ITT	PP	ITT	PP	ITT	PP	ITT	PP	ITT	PP
For calorie adequacy											
Visit 1	Visit 3	60.14	34.03	<0.001	<0.001	<0.001	<0.001	1	1	Yes	Yes
Visit 1	EOS	95.23	100.01	<0.001	<0.001	<0.001	<0.001	1	1	Yes	Yes
Visit 3	EOS	26.1	60.36	<0.001	<0.001	<0.001	<0.001	1	1	Yes	Yes
For protein intake											
Visit 1	Visit 3	8.49	21.81	0.0036	<0.001	0.0215	<0.001	1	1	Yes	Yes
Visit 1	EOS	80.77	91.01	<0.001	<0.001	<0.001	<0.001	1	1	Yes	Yes
Visit 3	EOS	63.47	62.13	<0.001	<0.001	<0.001	<0.001	1	1	Yes	Yes

Nutritional status

Nutritional status was assessed using the Subjective Global Assessment scale. It remained stable throughout the study, with all patients classified as well-nourished at all visits (ITT: *n* = 182, 100.0%; PP: *n* = 119, 100.0%). Despite surgical intervention, no decline in post-surgery nutritional status was observed among patients undergoing CABG.

Change in weight

A significant increase in weight was observed between baseline and EOS, and between Visit 1 (day of supplement initiation) and EOS, in both the ITT and PP groups. In the ITT group, the mean weight increased from 66.5 ± 9.9 kg at baseline to 66.8 ± 10.0 kg at EOS (*P* < 0.05). In the PP group, the mean weight increased from 64.9 ± 8.9 kg at baseline to 65.3 ± 9.1 kg at EOS (*P* < 0.05) (Table 4). The pairwise statistical comparison is provided in Table 5.

**Table 4:** Mean weight of the patients from baseline to the end of the study. Number of patients in the ITT group: *N* = 182, 100.0%; number of patients in the PP group: *N* = 119, 100.0% Baseline visit = Screening visit; Visit 1 = 24–48 hours post-surgery and initiation of the WPI-based oral nutritional supplement; Visit 3 = hospital discharge; and EOS = 30 days of supplementation post-discharge. EOS, end of study; ITT, intent-to-treat; PP, per-protocol; SD, standard deviation; WPI, whey protein isolate

Visit schedule	Patient groups			
	ITT, 182 (100%)		PP, 119 (100%)	
	Mean weight (kg)	SD	Mean weight (kg)	SD
Baseline	66.5	9.9	64.9	8.9
Visit 1	66.5	9.9	64.9	8.9
Visit 3	66.5	9.8	64.9	8.8
EOS	66.8	10.0	65.3	9.1

**Table 5:** Comparative assessment of weight across visits in the ITT (N = 182) and PP (N = 119) groups. Note: The degrees of freedom for the ITT group were 181, and for the PP group were 118. Paired t-tests were conducted to evaluate specific pairwise differences between study visits. Statistically significant differences were observed, particularly for comparisons between Baseline vs. EOS and Visit 1 vs. EOS, in both the ITT and PP populations. Statistical significance was determined based on a predefined alpha level of 0.05, with *P*-values < 0.05 considered significant and *P*-values ≥ 0.05 considered non-significant. Adjusted *P*-values were interpreted similarly, where adjusted significance (p.adj.signif) was denoted as *Yes* for *P* < 0.05 and *No *for *P* ≥ 0.05. Number of patients in the ITT group: *N* = 182, 100.0%; number of patients in the PP group: *N* = 119, 100.0%. Baseline visit = screening visit; Visit 1 = 24-48 hours post-surgery and initiation of WPI-based oral nutritional supplement; Visit 3 = hospital discharge; and EOS = 30 days of supplementation post-discharge. EOS, end of study; t, t-statistic; ITT, intent-to-treat; PP, per-protocol; df, degrees of freedom; CI, confidence interval (lower limit, upper limit); p.adj, adjusted P-value with Bonferroni's correction

Group 1	Group 2	ITT						PP					
		t	*P*-value	95% CI		p.adj	p.adj.significance (Yes/No)	t	*P*-value	95% CI		p.adj	p.adj.significance (Yes/No)
				Lower	Upper					Lower	Upper		
Baseline	Visit 1	-0.42	0.67	-0.12	0.08	1.00	No	-0.22	0.83	-0.17	0.14	1.00	No
	Visit 3	-0.09	0.93	-0.13	0.12	1.00	No	-0.18	0.86	-0.2	0.17	1.00	No
	EOS	-3.62	0.00	-0.44	-0.13	0.00	Yes	-3.27	0.00	-0.58	0.14	0.01	Yes
Visit 1	Visit 3	0.30	0.76	-0.09	0.13	1.00	No	0.00	1.00	-0.16	0.16	1.00	No
	EOS	-3.34	0.00	-0.42	-0.11	0.01	Yes	-3.09	0.00	-0.57	-0.12	0.03	Yes
Visit 3	EOS	-3.24	0.00	-0.45	-0.11	0.01	Yes	-2.76	0.01	-0.59	-0.097	0.07	No

Weight gain was observed in 60 (50.0%) patients in the PP group vs. 55 (30.0%) patients in the ITT group by EOS (Table 6).

**Table 6:** Descriptive statistics of the change in weight from baseline to the end of the study. Number of patients in the ITT group: *N* = 182 (100.0%); number of patients in the PP group: *N* = 119 (100.0%). Baseline visit = screening visit, and EOS = 30 days of supplementation post-discharge. ITT, intent-to-treat; PP, per-protocol

Change in weight	ITT (*N *= 182)		PP (*N* = 119)	
	n	%	n	%
Decrease (%)	29	16.0	21	18.0
No change (%)	98	54.0	38	32.0
Increase (%)	55	30.0	60	50.0

Hand-grip strength

The change in hand-grip strength was measured using an electronic hand dynamometer (CONSTANT, Model 14192-709E). Based on the recorded values, the instrument classifies patients into weak, normal, and strong categories according to predefined age- and sex-specific reference ranges. The cut-off values for each category according to the instrument are provided in Table 7.

**Table 7:** Cut-off values for different categories using a hand dynamometer.

Age (years)	Male			Female		
	Weak	Normal	Strong	Weak	Normal	Strong
18-19	˂35.7	35.7-55.5	>55.5	˂19.2	19.2-31.0	>31.0
20-24	˂36.8	36.8-56.6	>56.6	˂21.5	21.5-35.3	>35.3
25-29	˂37.7	37.7-57.5	>57.5	˂25.6	25.6-41.4	>41.4
30-34	˂36.0	36.0-55.8	>55.8	˂21.5	21.5-35.3	>35.3
35-39	˂35.8	35.8-55.6	>55.6	˂20.3	20.3-34.1	>34.1
40-44	˂35.5	35.5-55.3	>55.3	˂18.9	18.9-32.7	>32.7
45-49	˂34.7	34.7-54.5	>54.5	˂18.6	18.6-32.4	>32.4
50-54	˂32.9	32.9-50.7	>50.7	˂18.1	18.1-31.9	>31.9
55-59	˂30.7	30.7-48.5	>48.5	˂17.7	17.7-31.5	>31.5
60-64	˂30.2	30.2-48.0	>48.0	˂17.2	17.2-31.0	>31.0
65-69	˂28.2	28.2-44.0	>44.0	˂15.4	15.4-27.2	>27.2

At baseline, a substantial proportion of patients were categorised as weak in both groups (ITT: 94 (51.6%); PP: 43 (36.1%)), and this proportion increased by Visit 3 (on the day of discharge) (ITT: 130 (71.4%); PP: 85 (71.4%)). By EOS, the proportion of patients in the weak category declined significantly from Visit 3 (ITT: 71 (39.0%); PP: 38 (31.9%)) (*P* < 0.001). The percentage of patients in the normal category initially decreased from baseline to Visit 3 (ITT: 55 (30.2%) vs. 43 (23.6%); PP: 50 (42.0%) vs. 31 (26.1%)) but significantly increased by EOS from baseline and Visit 3 (ITT: 85 (46.7%); PP: 65 (54.6%)) (*P* < 0.001), with the change particularly evident in the PP group. The proportion of patients in the strong category declined sharply by Visit 3 (ITT: 9 (5.0%); PP: 3 (2.5%)) from baseline (ITT: 33 (18.1%); PP: 26 (21.9%)), with a significant increase noted at EOS as compared to Visit 3 (ITT: 26 (14.3%); PP: 16 (13.5%)) (*P* < 0.05) (Figure 3).

**Figure 3:**
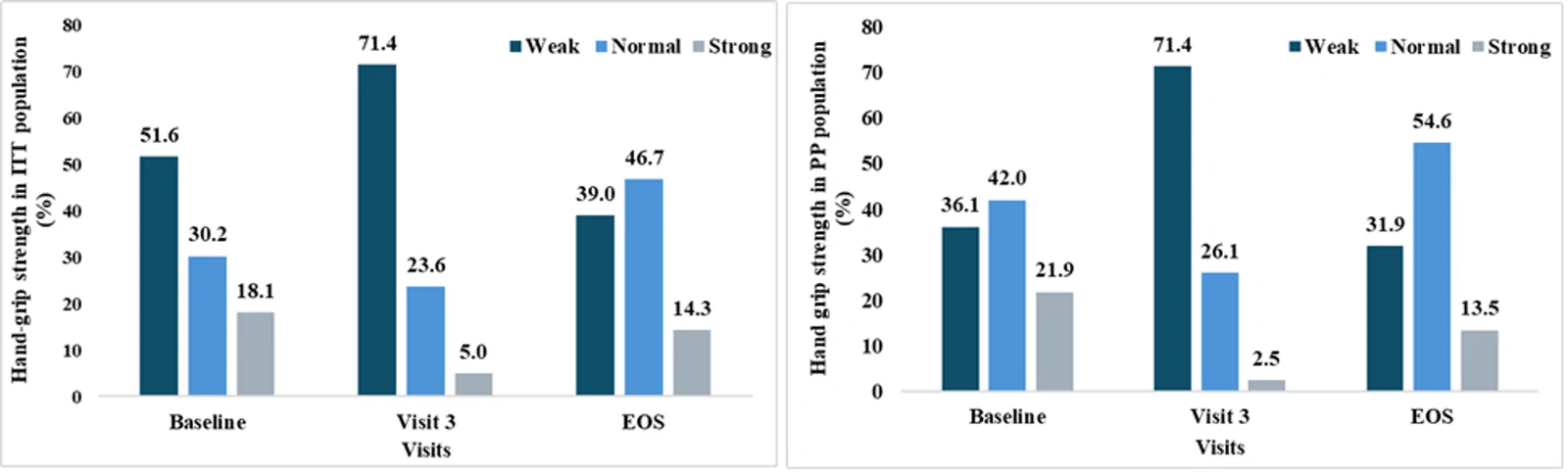
Summary of hand-grip strength across visits in the ITT (N = 182) and PP (N = 119) groups. Number of patients in the ITT group: *N* = 182 (100.0%); number of patients in the PP group: *N* = 119 (100.0%). Baseline visit = screening visit; Visit 3 = hospital discharge; and EOS = 30 days of supplementation post-discharge. The figure was prepared in Microsoft Excel, and pixels were adjusted using Microsoft Paint (Microsoft Corporation, Redmond, WA). EOS, end of study; ITT, intent-to-treat; PP, per-protocol

Transition analysis showed a statistically significant change between Visit 3 and EOS. A positive transition was observed in the normal (*P* < 0.001) and strong categories (*P* < 0.05). Many patients who were in the weak or normal category moved into the normal or strong category by EOS in both groups (*P* < 0.001) (Figure 4). The pairwise comparison is provided in Table 8.

**Figure 4:**
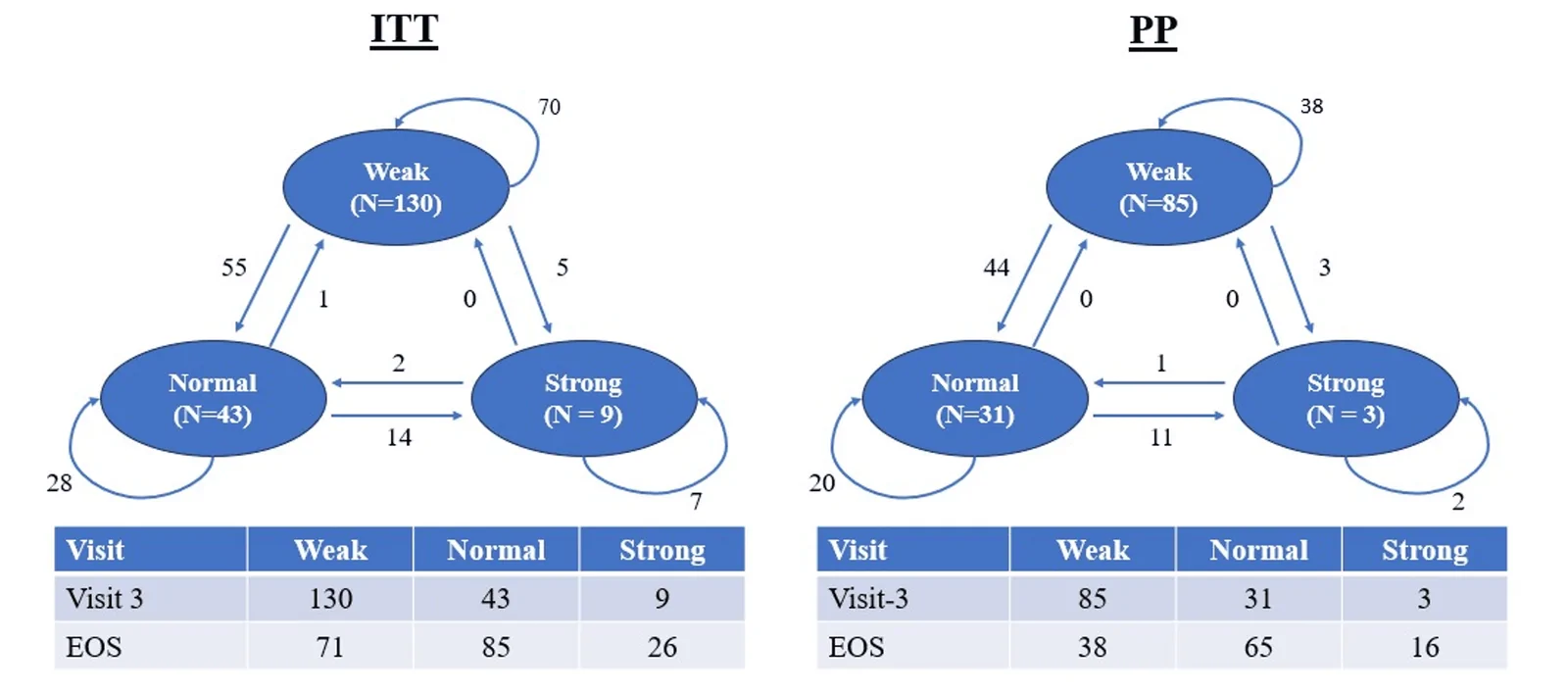
Transition in hand-grip strength from Visit 3 to end of study in the ITT (N = 182) and PP (N = 119) groups. Visit 3 = hospital discharge, and EOS = 30 days of supplementation post-discharge. Number of patients in the ITT group: *N* = 182 (100.0%); number of patients in the PP group: *N* = 119 (100.0%). The figure was prepared in Microsoft Word, and pixels were adjusted using Microsoft Paint (Microsoft Corporation, Redmond, WA). EOS, end of study; ITT, intent-to-treat; *N*, total number of patients; PP, per-protocol

**Table 8:** Comparative assessment of hand-grip transition category between visits in the ITT (N = 182) and PP (N = 119) groups. McNemar's test was applied to assess the statistical significance of changes in categorical outcomes over time based on paired observations. A *P*-value < 0.05 was considered indicative of a statistically significant change. Number of patients in the ITT group: *N* = 182 (100.0%); number of patients in the PP group: *N* = 119 (100.0%). Baseline visit = screening visit; Visit 3 = hospital discharge; and EOS = 30 days of supplementation post-discharge. EOS, end of study; ITT, intent-to-treat; PP, per-protocol; T1, first time point; T2, second time point

	Comparison	Both Yes		T1 Yes Only		T2 Yes Only		Both No		McNemar's statistic	*P*-value	Significant (Yes/No)
		n	%	n	%	n	%	n	%			
ITT group												
Weak	Baseline (T1) to EOS (T2)	57	31.3	37	20.3	14	7.7	74	40.7	9.4902	0.002	Yes
	Visit 3 (T1) to EOS (T2)	70	38.5	60	33.0	1	0.5	51	28.0	55.148	<0.001	Yes
Normal	Baseline (T1) to EOS (T2)	38	20.9	17	9.3	47	25.8	80	44.0	13.141	0.0002	Yes
	Visit 3 (T1) to EOS (T2)	28	15.4	15	8.2	57	31.3	82	45.1	23.347	<0.001	Yes
Strong	Baseline (T1) to EOS (T2)	15	8.2	18	9.9	11	6.0	138	75.8	1.2414	0.2652	No
	Visit 3 (T1) to EOS (T2)	7	3.8	2	1.1	19	10.4	154	84.6	12.19	0.00048	Yes
PP group												
Weak	Baseline (T1) to EOS (T2)	25	21.0	18	15.1	13	10.9	63	52.9	0.51613	0.4725	No
	Visit 3 (T1) to EOS (T2)	38	31.9	47	39.5	0	0.0	34	28.6	45.021	<0.001	Yes
Normal	Baseline (T1) to EOS (T2)	34	28.6	16	13.4	31	26.1	38	31.9	4.1702	0.041	Yes
	Visit 3 (T1) to EOS (T2)	20	16.8	11	9.2	45	37.8	43	36.1	19.446	<0.001	Yes
Strong	Baseline (T1) to EOS (T2)	9	7.6	17	14.3	7	5.9	86	72.3	3.375	0.06619	No
	Visit 3 (T1) to EOS (T2)	2	1.7	1	0.8	14	11.8	102	85.7	9.6	0.0019	Yes

Patient compliance and experience with WPI-based nutritional supplement

All patients in both the ITT and PP groups reported a “good” or “great” experience (ITT: 182 (100.0%); PP: 119 (100.0%)) with the WPI-based nutritional supplement at Visit 3 and EOS and were compliant with the supplement prescription (ITT: 182 (100.0%); PP: 119 (100.0%)). Nearly all patients (ITT: 181 (99.4%); PP: 119 (100.0%)) liked the taste of the supplement and reported no use-related difficulty (ITT: 182 (100.0%); PP: 119 (100.0%)).

Physician experience with WPI-based nutritional supplement

At Visit 3 and EOS, the physicians rated the experience as *good *or *great* for all patients (ITT: 182, 100.0%; PP: 119, 100.0%). Based on physician experience, muscle strength recovered in the ITT group (181, 99.4%) and the PP group (118, 99.2%) by EOS.

Safety assessment

No adverse events related to the WPI-based nutritional supplement were reported during the study period. One death occurred during the study period and was deemed unrelated to the WPI-based nutritional supplement by the investigator.

## Discussion

The current study assessed the effects of early postoperative nutrition with a WPI-based oral supplement on patients undergoing CABG surgery. The study was conducted in routine clinical practice, where WPI-based supplementation and dosage were investigator-determined based on each patient's nutritional status, oral intake, and estimated requirements. Protocol-defined visits and assessments were used only for structured data collection and did not involve randomisation, comparator allocation, or study-mandated treatment assignment. The study findings indicated an increase in calorie and protein intake, weight, and muscle strength recovery. The study outcomes highlight the integration of WPI-based oral nutritional supplements into standard postoperative recovery regimens. These results align with previous research demonstrating the benefits of postoperative intake of whey protein on clinical and nutritional outcomes [28-30].

Early postoperative nutritional intake is critical for enhancing recovery post-surgery, as it reinforces stress responses and mitigates muscle protein catabolism. It is also associated with reduced infectious complications, enhanced wound healing, improved functionality, decreased ICU admissions, and shorter hospitalisation [16,31]. Calorie and protein intake are often inadequate to meet the perioperative metabolic demands during the catabolic phase. Therefore, oral nutrition supplements are an integral component of nutritional management, helping maintain muscle mass and attenuating protein catabolism [15,32].

Cardiac surgery patients frequently do not meet their daily protein requirements, primarily attributable to postoperative catabolism, thereby necessitating targeted supplementation regimens in routine clinical practice. Therefore, nutritional supplementation may help support the maintenance of nutritional status and contribute to postoperative recovery as part of routine clinical care. Additionally, ESPEN and ERAS guidelines recommend caloric and protein goals for patients recovering from surgery to improve nutritional status and recovery [2,9-11]. Consistent with these recommendations, initiation of WPI-based nutritional supplementation within 24-48 hours after surgery was associated with an increase in calorie and protein adequacy, along with other nutritional outcomes, in postoperative patients.

The biochemical properties of WPI make it an ideal supplement to stimulate muscle protein synthesis and improve postoperative outcomes [16,33-35]. A randomised controlled trial (RCT) involving patients with congestive heart failure (*n* = 20) evaluated the effects of an oral nutritional supplement containing whey peptides enriched with BCAAs as part of a cardiac rehabilitation programme. Supplementation with the whey peptide formulation resulted in significant improvements in clinical outcomes, including increased fat-free mass, enhanced energy capacity, higher peak oxygen uptake, and improved six-minute walk test performance compared with conventional rehabilitation alone, thereby demonstrating the efficacy of whey peptide supplementation in cardiac rehabilitation [36].

The association between whey protein supplementation and improved nutritional status has been probed in multiple studies [32,37]. The nutritional targets for patients aligned with the ESPEN guideline were 25-30 kcal/kg/day of calories and 1.1-1.5 g/kg/day of protein from the oral diet and a WPI-based nutritional supplement. In our study, participants achieved adequate calorie and protein intake, with significant improvements from Visit 1 to EOS within 24-48 hours post-surgery, particularly in the PP group. Observational studies supporting these outcomes indicate that early postoperative nutrition can improve clinical outcomes by reducing the length of hospital stay, minimising perioperative complications, and supporting enhanced recovery [38-40]. A multicentric prospective observational study among institutionalised adults (*n* = 91) reported improved nutritional status - evidenced by weight gain, increased BMI, and higher Mini Nutritional Assessment scores - following a 12-week intervention combining protein-rich oral supplements and physical exercise [41,42]. A post hoc analysis of an RCT conducted in neurocritical patients (*n* = 116) showed significantly higher protein intake (1.7 g/kg/day) in those receiving whey protein supplementation compared with controls [43].

Malnutrition is a modifiable risk factor in both the pre- and postoperative periods, and appropriate nutritional support can significantly improve clinical outcomes [15,44]. In the present study, patients' nutritional status was maintained despite surgical intervention. A recent meta-analysis of ten global studies highlighted the positive impact of protein supplementation on body composition and functional recovery in cardiac rehabilitation patients [45]. Similarly, a single-centre, pragmatic, parallel-group RCT in malnourished patients with cancer undergoing chemotherapy (*n* = 166) reported improvements in body composition, muscle strength, and body weight along with reduced toxicity after three months of WPI supplementation [46]. Additionally, a double-blind RCT among patients undergoing one-anastomosis gastric bypass (*n* = 78) demonstrated that whey protein supplementation significantly supported early postoperative muscle mass retention [47].

In our study, weight gain was observed in both the ITT and PP groups, with 60 (50.0%) of patients in the PP group exhibiting weight gain at EOS. Although the observed weight gain was modest, it may be clinically relevant in the early postoperative CABG setting, where catabolism and weight loss are common. Therefore, body weight maintenance or slight weight gain may be considered favourable during recovery. Muscle strength, assessed by hand-grip strength, also improved over the study period. Although a transient decline in hand-grip strength was observed in the immediate postoperative phase, the significant increase at EOS was noted, suggesting muscle recovery. These findings align with a recent meta-analysis of RCTs that reported a significant improvement in hand-grip strength at three months post-intervention (mean difference = 3.11 (95% CI: 1.45-4.78)). The interventions included leucine-rich whey protein, further validating the efficacy of protein-based nutritional strategies in enhancing muscle function [48].

To the best of our knowledge, this is the first prospective study to describe the use of WPI-based oral nutritional supplementation for supporting postoperative nutritional outcomes and muscle strength recovery in patients undergoing CABG in the Indian clinical context. Gradual recovery after CABG occurs over time and may be influenced by routine oral diet, postoperative care, and nutritional supplementation. Therefore, the study findings should be interpreted with caution, as the single-arm design does not allow the observed outcomes to be attributed solely to WPI-based supplementation. Although the study sponsor was the manufacturer of the nutritional supplement used in the study, it had no role in patient selection, data management, data analysis, or interpretation of the study results. The analysis was conducted independently by the study investigators.

Limitations

Despite providing substantial information on the use of a WPI-based nutritional supplement in post-surgical patients, the current analysis has certain limitations. The current study was an RWE study in which patients were selected based on routine clinical practice. Since the decision to recommend WPI-based supplementation was based on investigator assessment in routine clinical practice rather than a fully standardised eligibility algorithm, potential selection bias cannot be excluded. Patients who were unable to meet their daily dietary protein requirement were included. However, this approach might not be representative of all post-surgical patients. Patients with BMI >30 kg/m² were excluded from the study, limiting the generalisability of the study findings. In particular, exclusion of patients with obesity may limit applicability of the results to the broader CABG population, as obesity is common in this patient group. Additionally, no adjustment for confounding was done within the study. The observational single-arm design and absence of multivariable adjustment for potential confounders, including age, sex, diabetes status, baseline BMI, nutritional status, and site-specific factors, should be considered when interpreting the findings. These factors may have influenced nutritional recovery and functional outcomes independently of nutritional supplementation. Another limitation pertains to patient follow-up. Due to the real-world nature of the study, consistent follow-up for the EOS visit was challenging. Approximately 40.5% of patients did not adhere to the scheduled study visits. These patients either visited early or after the predefined timelines and were included in the ITT group. Because this was a single-arm study without a concurrent or historical comparison group, the observed changes cannot be definitively attributed to WPI-based nutritional supplementation. Routine postoperative recovery, standard dietary care, and other clinical factors may also have contributed to the observed improvements. Therefore, the findings should be interpreted as temporal changes during postoperative follow-up, and randomised or well-controlled comparative studies are warranted to confirm the independent effect of WPI-based supplementation in patients undergoing CABG. Additionally, several clinically relevant perioperative and baseline variables, including CABG type, smoking status, ejection fraction, number of grafts, ICU stay, length of hospital stay, detailed baseline calorie/protein intake, and site-level differences, were not part of the predefined study objectives and were not systematically collected. Therefore, their potential influence on nutritional and functional outcomes could not be assessed. Further randomised comparative studies are warranted to confirm the effects of WPI supplementation in post-surgical patients. Additionally, this study was funded by Otsuka Pharmaceutical India Private Limited, which also provided the study product. Sponsor representatives were co-authors and contributed to study conception, design, and manuscript preparation. The sponsor had no role in data collection, analysis, or interpretation; however, its involvement represents a potential source of bias and should be considered when interpreting the findings.

## Conclusions

This Indian real-world study in post-surgical patients highlights the importance of early initiation and continued use of WPI-based high-protein supplementation for at least 30 days post-surgery. The study demonstrated that WPI-based oral supplementation effectively supported the achievement of recommended caloric (25-30 kcal/kg/day) and protein (1.1-1.5 g/kg/day) intake targets and played a key role in promoting weight gain, enhancing muscle strength recovery as assessed by hand-grip strength, and maintaining a *well-nourished *status throughout the study period. Overall, these findings strongly support integrating WPI-based oral nutritional supplementation into routine postoperative care to promote nutritional resilience and accelerate muscle strength recovery in cardiac surgery patients.

As patients received WPI-based supplementation within a broader nutritional care framework that included dietitian follow-up, nutritional counselling, and routine postoperative care, the observed outcomes cannot be attributed solely to the supplement. The findings should therefore be interpreted as reflecting the combined effect of nutritional supplementation and overall postoperative nutritional care.
